# Attraction of Cohesion in Gold Fillings

**Published:** 1848-07

**Authors:** J. W. Crane

**Affiliations:** Dentist, New York.


### ARTICLE IX.

### *Attraction of Cohesion in Gold Fillings.* By J. W. Crane, M. D., Dentist, New York.

Although much has been said and written cn the subject of filling teeth, yet there is room for argument and instruction for al!. When a tooth is well filled, we must admit that the cavity is perfectly dry, free from decay, and will continue so, under ordinary circumstances, for a number of years. But the manner of accomplishing this, has been a subject of discussion among the most respectable members of the dental profession. It is not necessary to enumerate the various modes of performing this operation, but we may safely say that each one thinks his own way the best, or he would relinquish it and adopt another. There is a test on almost every subject, by which we may arrive at the truth, and if one can be discovered, well adapted to this subject, we shall not hesitate to apply it.

There is one point concerning a gold filling, which is beyond controversy, and that is, a perfect solid mass of gold, well adapted to every part of the cavity, so that air and water are entirely excluded, is the most valuable filling. If gold in a fusible state could be poured into the cavity of a tooth, we should conclude that all the little irregularities would be filled much more perfectly than they could be in the ordinary way. All kinds of metal in a solid state are said to remain so by the attraction of cohesion. Earthy matter is capable of being kept together by the same principle.

But, since gold cannot be used in a fusible state, the question is, can we produce this attraction by any mechanical power. Philosophy teaches us, that if two pieces of metal are made to touch each other, they will unite by the attraction of cohesion. It also teaches, that if a vapor passes between them, at the time they are supposed to be in contact, they do not touch each other.

Take for example two pieces of lead, and cut a clean surface on each, and press them hard together with a slight turn, and you will unite them so that it will require considerable force to separate them. But if you pass your finger over the surface of either, or breathe on them, the attractive power is destroyed. Now I propose to show that this power has been applied to the operation of filling teeth, and is practicable in all cases where repulsion can be overcome.

It may not be so perfect in all parts, as that which is produced by fusion, but I am credibly informed that the same mass if melted, would fill the same space, because one is annealed, and the other hardened.

To accomplish this, it is necessary to cut through the filling in every direction, and the surfaces thus cut, united by the power of the wedge. To prevent any delay, (after the cavity is perfectly cleansed,) the gold should be slightly twisted into a cord or thread of sufficient length to fill the tooth, and taken up with the point of the instrument as it is wanted, taking care that no moisture or even the breath shall come in contact with the gold.

In 1829, I commenced filling teeth on the above mentioned plan, with a spring tempered excavator, and the only difficulty attending the operation has been to overcome the power of repulsion. The filling instruments should be curved and straight so as to cut in every direction, and should be kept sharp like the excavator, and should be spring temper, firmly fixed in handles. They should be thrust deep into the gold, carrying in a quantity of the metal at every thrust, until the cavity is more than full. The filling forceps, file, burnisher, &c. will complete the operation.

